# A Case of Deep Vein Thrombosis Complicated by Right Atrial Clot-in-Transit and Pulmonary Embolism in an Alcoholic Male

**DOI:** 10.7759/cureus.73561

**Published:** 2024-11-12

**Authors:** Tamer Zahdeh, Mahmoud El Hajj, Aiswarya Nair, Silpa Choday

**Affiliations:** 1 Internal Medicine, Montefiore St. Luke's Cornwall, Newburgh, USA; 2 Internal Medicine, Creighton University School of Health Science, Omaha, USA

**Keywords:** acute pulmonary embolism, alcohol use, deep venous thrombosis, dvt, hemodynamic instability, obstructive shock, pe, pulmonary thrombectomy, right atrial thrombus, thrombectomy

## Abstract

This report presents the case of a 62-year-old male with a history of chronic alcohol abuse who developed deep vein thrombosis (DVT) complicated by extensive bilateral pulmonary embolism (PE) and a right atrial thrombus. The presence of a right atrial thrombus in conjunction with PE and DVT is a rare and serious clinical presentation, often associated with a high thrombotic burden and increased risk of mortality. The patient initially presented with worsening shortness of breath following an occupational injury that resulted in a left heel laceration, subsequently leading to a significant thromboembolic event. The clinical course was marked by syncope and hemodynamic instability, necessitating urgent intervention. Initial management involved anticoagulation; however, due to the patient’s critical condition and hemodynamic instability, urgent mechanical aspiration thrombectomy was performed, successfully removing the thrombi and stabilizing the patient. This case stresses the multifactorial nature of thromboembolism, highlighting the interplay between trauma, chronic alcohol abuse, and thrombus formation. The report also emphasizes the importance of considering lifestyle and occupational factors in the risk assessment and management of thromboembolic events, while also illustrating the efficacy of advanced interventional techniques in treating complex cases of PE with intracardiac thrombi.

## Introduction

Deep vein thrombosis (DVT) and pulmonary embolism (PE) represent significant and often interrelated vascular emergencies, marked by the formation of thrombi within the deep veins and their subsequent migration to the pulmonary arteries. The clinical spectrum of these conditions can range from asymptomatic cases to life-threatening situations, particularly when complicated by right atrial thrombus, as seen in this case. PE is a critical condition with an incidence rate of 60 to 70 cases per 100,000 individuals annually in the United States, posing a substantial public health burden due to its associated morbidity and mortality [1].

This report describes the case of a 62-year-old male with a history of chronic alcohol use and achalasia, presenting with a severe clinical course of DVT complicated by extensive bilateral PE and right atrial thrombus. The patient's symptoms began following an occupational injury leading to a laceration and subsequent infection of the left heel. The clinical presentation included progressive dyspnea, syncope, and hemodynamic instability, necessitating urgent medical intervention. Initial laboratory findings were indicative of an inflammatory response and myocardial stress, as evidenced by elevated troponin I and brain natriuretic peptide (BNP) levels, alongside leukocytosis.

The pathophysiological interplay between trauma, alcohol use, and thromboembolic events is multifaceted. Trauma, particularly to the lower extremities, can trigger the formation of DVT through endothelial injury, venous stasis, and hypercoagulability, collectively known as Virchow's triad [2]. Chronic alcohol use may further exacerbate the risk of thrombosis by affecting coagulation pathways, despite its complex and paradoxical effects on hemostasis [3]. This case underscores the importance of considering both lifestyle and occupational factors in the risk assessment for thromboembolic events.

Management of this case required a multidisciplinary approach, highlighting the challenges in treating patients with hemodynamically unstable PE and concomitant right atrial thrombus. The standard treatment regimen includes anticoagulation; however, this patient’s critical condition warranted the use of mechanical aspiration thrombectomy, an advanced intervention facilitated by interventional radiology [4,5]. This approach was crucial in achieving rapid clot removal and stabilization of the patient’s hemodynamic status.

This case report aims to provide a comprehensive overview of the clinical presentation, diagnostic evaluation, and management strategies for a patient with DVT complicated by PE and right atrial thrombus in the context of chronic alcohol use. The discussion will delve into the implications for clinical practice, emphasizing the need for vigilance in patients with atypical risk factors and the potential benefits of advanced interventional techniques in acute settings.

## Case presentation

A 62-year-old White male presented with a past medical history of achalasia, for which he underwent laparoscopic Heller Myotomy in 2019, and chronic alcohol abuse (five cans of beer daily for 40 years) without a known liver disease presented with complaints of worsening shortness of breath. The patient reported that a week before his admission, he had an occupational injury where he accidentally lacerated the posterior aspect of his left heel. On the following day, the patient developed progressively worsening pain in the heel that restricted his physical activity and ability to ambulate. His pain became worse to the point that he had to crawl on the floor to get around in his house. Two days after the initial injury, while crawling to the restroom, he suddenly lost consciousness. Upon regaining consciousness, he found himself on the floor experiencing severe dyspnea. He added that on the following morning, after crawling to the kitchen for water, he became severely dyspneic upon returning to the couch. His symptoms continued to worsen until the day of his admission when he passed out yet again, prompting him to call emergency medical services. Upon taking further history, the patient also reported noticing an orange-colored urine with mild burning sensation two days prior to his admission. However, he denied further symptoms such as loss of appetite, fever, chills, chest pain, cough, palpitations, headaches, visual changes, or gastrointestinal complaints. The patient endorsed working in an office job as a building operation engineer and having an overall sedentary lifestyle. He also endorsed drinking roughly five beer cans every night for the last 40 years. He was an ex-smoker with a five pack-year history, having quit 10 years ago. He denied using illicit drugs or recent travel history. His family history is notable for diabetes mellitus and gout in his father, varicose veins in his mother, and pancreatic cancer secondary to alcohol abuse in his older brother.

**Figure 1:**
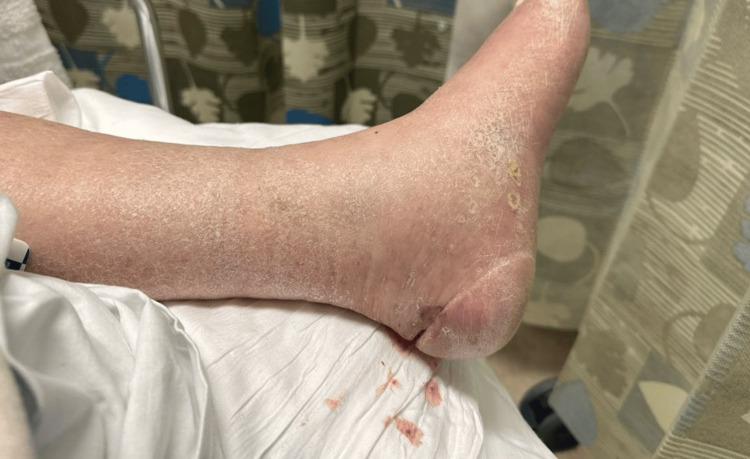
Horizontal laceration of the posterior left heel with lower extremity edema.

In the emergency department (ED), the patient was alert, awake, and oriented. Initial vital signs were remarkable for a heart rate of 115 beats/min, blood pressure of 160/90 mmHg, respiratory rate of 24 breaths/min, normal temperature of 97.0°F, and oxygen saturation of 98% in room air. On physical examination, the patient was an overweight (body mass index of 29 kg/m^2^) diaphoretic male. Vital signs showed tachycardia (115 beats/min) and tachypnea (24 breaths/min). He had grade +1 bilateral lower extremity pitting edema, more pronounced and tender on the left. A 3-cm horizontal laceration was observed on the left heel, with severe surrounding tenderness to palpation (Figure 1). Cardiovascular and respiratory examinations were otherwise unremarkable.

Initial workup in the ED was notable for leukocytosis (white blood count of 11.86K cells/μL), elevated troponin I - high sensitivity (148.4 ng/L), elevated brain natriuretic peptide (3303 pg/mL), and elevated lactic acid (3.9 mmol/L), and electrocardiogram (ECG) showed sinus tachycardia with non-specific ST and T wave abnormalities. The patient was then deemed to have sepsis in the setting of suspected cellulitis, for which blood cultures were collected and the patient was given a bolus of 2 liters of isotonic saline as well as one dose of intravenous (IV) vancomycin 1 gram and IV piperacillin/tazobactam 4.5 grams. Chest X-ray was unremarkable, and left foot X-ray showed soft tissue swelling and diffuse degenerative changes. A few hours later, the patient's blood pressure dropped to 73/53 mmHg and oxygen saturation dropped to 88% in room air, for which the patient was given another bolus of 2 liters isotonic saline and placed on continuous nasal cannula oxygen therapy. Repeat troponin and lactic acid both trended up to 270.2 ng/L and 4.3 mmol/L, respectively. Computed tomography (CT) of the left foot with IV contrast showed no evidence of fracture, dislocation, or osteomyelitis. The patient was then admitted to the step-down unit (SDU) for further workup and management.

**Figure 2:**
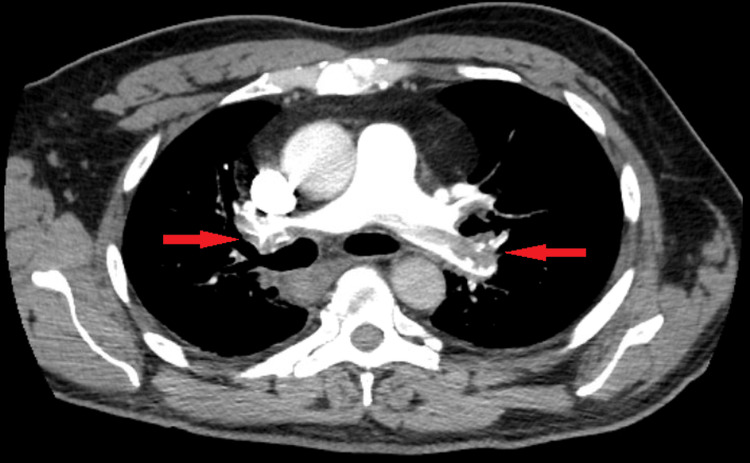
CT angiography of the chest showing bilateral filling defects (red arrows) in the main pulmonary arteries and their tributaries.

**Figure 3:**
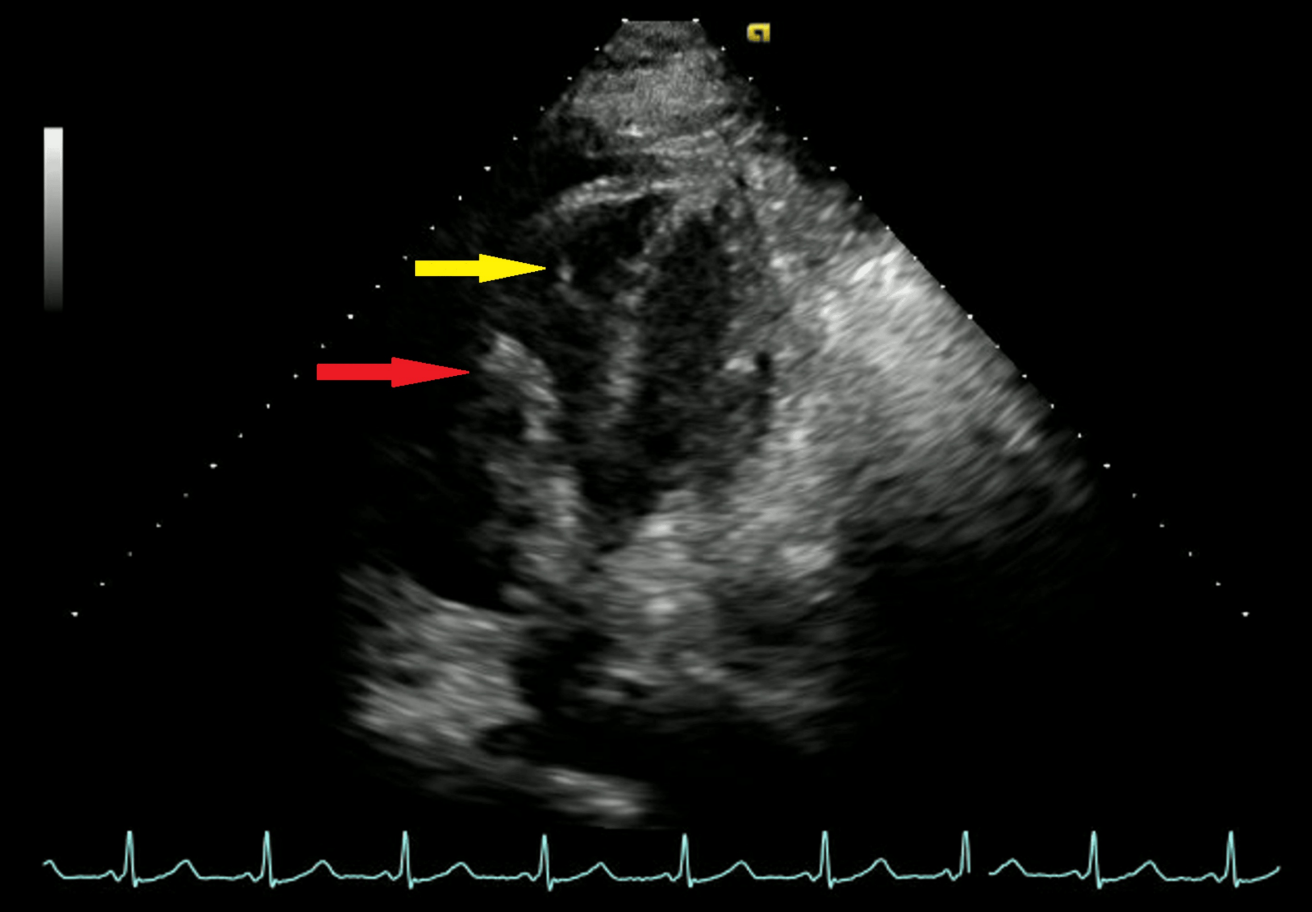
Apical view of transthoracic echocardiogram showing a right atrial thrombus-in-transit (red arrow) extending into the right ventricle (yellow arrow).

On route to SDU, the patient had a syncopal episode, raising a concern for PE. CT angiography (CTA) of the chest and lower extremity Doppler were then ordered stat. CTA revealed extensive pulmonary emboli within the right and left pulmonary arteries, upper lobes bilaterally, right middle lobe, and lower lobes bilaterally (Figure 2), as well as minimal bilateral subsegmental atelectasis or lung scarring. No thoracic aortic aneurysm was identified. There was no lung consolidation, endobronchial mass, or pleural effusion. There was prominence of interstitial markings in both lungs, likely due to chronic interstitial lung disease. There was no neoplastic hilar or mediastinal adenopathy. The heart was not enlarged. There was dilatation of the thoracic esophagus, extending from the proximal thorax to the gastroesophageal junction, suspicious for gastroesophageal reflux. Lower extremity Doppler also revealed a partially occlusive thrombus within the left popliteal vein and the calf veins bilaterally. Subsequently, the patient was started on heparin infusion, and interventional radiology was consulted urgently in the setting of hemodynamic instability. Echocardiography was performed, which showed normal left ventricular size albeit smaller than the right ventricular chamber with hyperdynamic systolic function and ejection fraction of 70%, moderately dilated right ventricle with global hypokinesis and septal flattening consistent with right-sided pressure and volume overload, moderately dilated right atrium containing a heterogenous and highly mobile echo-density within the atrium extending into the right ventricle consistent with a thrombus (Figure 3), severe pulmonary hypertension with right ventricular systolic pressure of 75 mmHg, moderate tricuspid regurgitation, and distended inferior vena cava without evidence of collapse.

**Figure 4:**
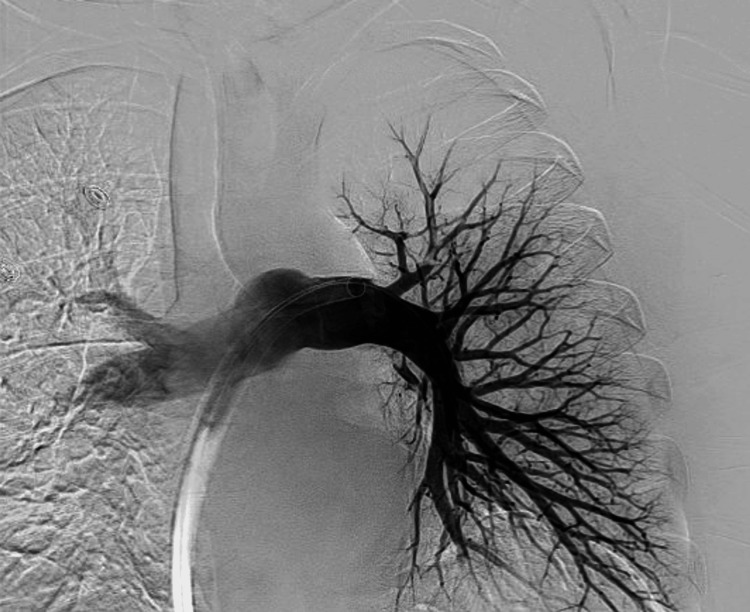
Intra-procedural angiogram following thrombectomy showing considerable improvement with good flow to all lobes in all segments with no significant residual clot.

**Figure 5:**
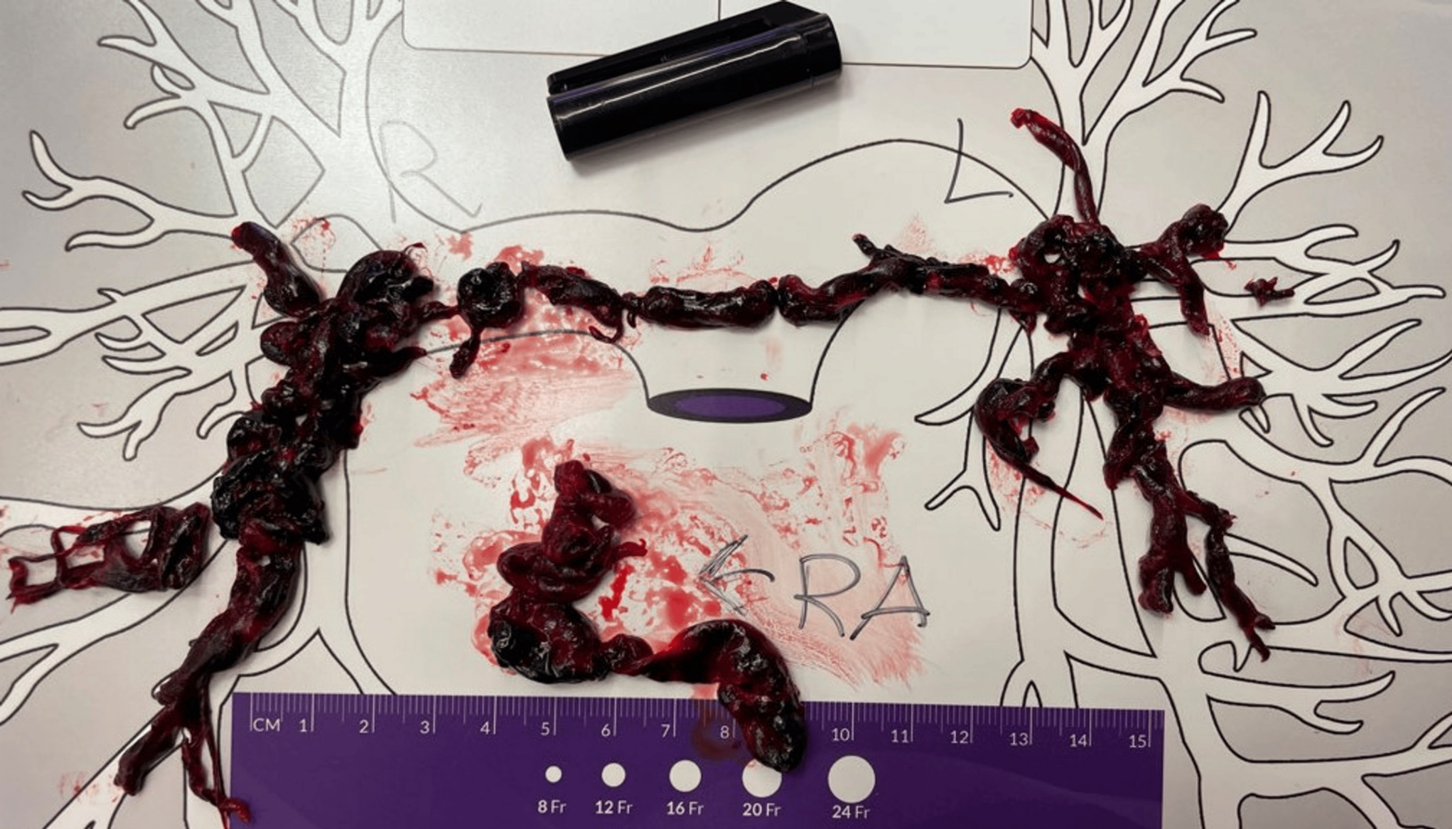
Gross photo of the clots retrieved following thrombectomy.

The patient was urgently evaluated by the on-call interventional radiologist who recommended urgent thrombectomy in light of the patient's hemodynamic instability. He then underwent successful mechanical aspiration thrombectomy (using the T24 Inari FlowTriever® device, Inari Medical, Inc., located in Irvine, CA) of right atrial clot-in-transit using real-time transthoracic echocardiogram guidance as well as successful bilateral pulmonary arterial mechanical thrombectomy/extirpation with significant clot clearance from the main and lobar pulmonary arteries and subsequent excellent flow to the bilateral pulmonary arteries on final angiography (Figures 4, 5).

The patient was then moved to SDU and placed back on IV heparin drip. He was also continued on IV cefazolin 1 gram three times daily per the infectious diseases doctor's recommendations. The patient was also evaluated by the podiatrist who left the wound to heal as second intention and ordered magnetic resonance imaging (MRI) of the left ankle to rule out Achilles tendon laceration near its insertion, which showed diffuse circumferential subcutaneous edema without evidence of an abscess and small volume retrocalcaneal bursitis in the setting of mild Achilles tendinosis without evidence to support an infected/torn Achilles tendon, fractures, or osteomyelitis. On the following day, the patient's vital signs stabilized within the normal limits, and he was gradually weaned off the oxygen treatment. He was then evaluated by the hematologist who recommended anticoagulation treatment for at least one year with apixaban. The patient was therefore started on a loading dose of apixaban of 10 mg twice daily for seven days with instructions to take apixaban 5 mg twice daily thereafter. The patient tolerated the anticoagulant well without bleeding or a drop in hemoglobin. He was instructed on discharge to undergo repeat CTA of the chest and echocardiography within four to six weeks.

## Discussion

The co-occurrence of a right atrial or ventricular thrombus with PE secondary to DVT is a relatively rare but serious clinical presentation. Such cases are particularly concerning due to the potential for severe hemodynamic compromise and increased risk of mortality. The incidence of right atrial thrombus in patients with PE varies but is generally considered to be uncommon. It is often associated with massive PE, and, in many cases, the thrombus may be detected incidentally during imaging studies conducted for other reasons [6,7].

The presence of a right atrial thrombus in the context of PE suggests a high thrombotic burden, potentially indicating an embolic source extending from the lower extremities to the pulmonary circulation. According to Moser et al., right atrial thrombi may be present in up to 4% of patients with acute PE, though this number can vary depending on the population studied and the imaging modalities used [2]. These thrombi are often described as "thrombi in transit," indicating that they are on the path from the systemic venous system to the pulmonary arteries. This condition is critical, as the thrombus can lead to acute right heart failure, a significant cause of mortality in PE patients [6,7].

In the case presented, the detection of a right atrial thrombus, alongside extensive bilateral PE and DVT, stresses the severity of the patient’s condition. The patient's symptoms of syncope and hemodynamic instability, as well as the findings of right ventricular dilation and pressure overload, align with the severe nature of thromboembolic disease involving the right atrium and ventricle [6,7].

Trauma, particularly to the lower extremities, is a well-recognized risk factor for the development of DVT. This is largely due to the elements of Virchow’s triad: endothelial injury, venous stasis, and a hypercoagulable state. Endothelial injury, as in the case of a laceration or crush injury, exposes subendothelial tissue and triggers a cascade of coagulation processes that can lead to thrombus formation [8]. In this case, the patient's occupational injury, which led to a laceration of the left heel, likely initiated a local inflammatory response that contributed to thrombus formation. The subsequent development of cellulitis could have exacerbated the inflammatory and hypercoagulable state, further increasing the risk of thromboembolism.

Chronic alcohol use is another critical yet complex factor in the development of thromboembolic events. While moderate alcohol consumption has been associated with a decreased risk of venous thromboembolism [9], chronic heavy drinking may increase this risk. The relationship between alcohol intake and thrombotic risk appears to follow a J-shaped curve, with both abstainers and heavy drinkers at higher risk compared to moderate drinkers. Chronic alcohol abuse can lead to liver disease, which is associated with coagulopathy and an increased risk of thrombosis due to altered levels of coagulation factors [10]. In this patient, the long-standing history of consuming four to five beers nightly likely contributed to a complex coagulation profile, exacerbating the risk of DVT and subsequent PE. The exact mechanisms by which alcohol influences thrombogenesis are multifaceted, involving platelet function, fibrinolysis, and changes in plasma levels of fibrinogen and other clotting factors [10,11].

A review of similar case reports highlights the variable presentations and outcomes of patients with concomitant DVT, PE, and intracardiac thrombi. For instance, the presence of a right atrial thrombus often portends a worse prognosis due to the potential for massive PE and sudden hemodynamic collapse. In a case series by Torbicki et al., patients with right atrial thrombi demonstrated higher mortality rates, particularly if the thrombus was mobile or extended into the right ventricle [6]. This is consistent with the current case, where the patient exhibited severe symptoms and required urgent mechanical thrombectomy.

Comparatively, the role of lifestyle factors such as alcohol use in thromboembolism has been discussed in several studies. Mukamal et al. reported that moderate alcohol consumption might have a protective effect against DVT, but excessive intake, as seen in chronic abuse, is associated with an increased risk [11]. This dichotomy reflects the complex interplay between alcohol and coagulation and suggests that patient education on lifestyle modifications could be a crucial component of DVT and PE prevention strategies [12].

## Conclusions

In this report, we presented the case of a 62-year-old male with a complex presentation of DVT complicated by extensive PE and a right atrial thrombus, against the backdrop of chronic alcohol use and recent trauma. The patient's acute symptoms of severe dyspnea, syncope, and hemodynamic instability highlighted the urgent nature of his condition, necessitating a multidisciplinary approach including advanced diagnostic imaging and mechanical thrombectomy. The presence of a right atrial thrombus, although rare, indicated a significant thromboembolic burden and underscored the risk of rapid clinical deterioration. This case illustrates the considerable role of trauma-induced endothelial injury and chronic alcohol use in exacerbating the risk of thromboembolic events, as both factors can contribute to the conditions outlined in Virchow’s triad. The successful intervention, including mechanical thrombectomy, emphasizes the importance of advanced therapeutic strategies in managing massive PE and intracardiac thrombi. A review of similar cases in the literature reinforces the critical need for early recognition and prompt treatment of such severe thromboembolic events. Furthermore, this case underscores the necessity of addressing modifiable risk factors, such as alcohol consumption, highlighting the need for a holistic approach to patient management and prevention strategies in clinical practice.
